# Investigation of the Method for Measuring the Surface Property Parameters of Variable Charge Minerals Using Ion Selection Electrode

**DOI:** 10.3390/ma17236012

**Published:** 2024-12-09

**Authors:** Jiaqi Sun, Xinmin Liu, Hang Li, Deyuan Ma

**Affiliations:** Chongqing Key Laboratory of Interface Process and Soil Health, College of Resources and Environment, Southwest University, Chongqing 400715, China; 15621322882@163.com (J.S.); lihangswu@163.com (H.L.); madeyuan137@163.com (D.M.)

**Keywords:** variable charge minerals, ion-selective electrode method, surface property parameters, specific surface area, surface potential

## Abstract

In this study, the surface property parameters of non-swelling variable charge minerals, kaolinite and goethite, were determined using the ion-selective electrode method. The effects of experimental conditions, such as pH, ion concentration ratio, and liquid addition method, on the measurement results were clarified to provide a reference for accurately assessing the surface properties of variable charge materials. The research employed ion adsorption equilibrium experiments under varying pH levels, ion concentration ratios, and liquid addition methods. A combined surface property analysis was conducted using K^+^ and Ca^2+^ as indicator ions to characterize surface parameters. The results were compared with the specific surface area obtained via the BET method to verify accuracy, thereby identifying optimal measurement conditions. The study led to the following five conclusions. (1) pH significantly affected the adsorption amount and ratio of indicator cations, thereby influencing the accuracy of surface property parameters. (2) The addition method and concentration ratio of electrolytes influenced the measurement accuracy by affecting the adsorption state and equilibrium time of the two indicator cations. (3) For kaolinite, the optimal initial pH ranged from 7.5 to 8.5 in the KOH + Ca(OH)_2_ system and from 8.0 to 8.5 in the KOH + CaCl_2_ system, while the equilibrium pH was 7.5 to 8.0 in both systems. The optimal ion concentration ratios were c_K_:c_Ca_ = 2:1 and 9:1, respectively. (4) For goethite, the optimal initial and equilibrium pH values were 8.5 to 9.0 and 7.5 to 8.0, respectively, in both KOH + Ca(OH)_2_ and KOH + CaCl_2_ systems. The optimal ion concentration ratios were 4:1 and 20:1, respectively. (5) Through comparison, the optimal initial pH for measuring the two variable charge minerals was determined to be 8.5 ± 0.1, with the optimal equilibrium pH at 7.5 ± 0.1. However, the concentration ratios varied significantly, suggesting the need for systematic research by adjusting a series of ion concentration ratios based on the initial pH.

## 1. Introduction

Surface property parameters, including surface potential, specific surface area, surface charge density, surface charge quantity, and surface electric field strength, play a crucial role in reactions at the soil solid–liquid interface. These parameters control interactions among soil particles, which influence the formation, stability, and dispersion of soil aggregates. Consequently, they significantly impact macroscopic soil properties such as water retention, nutrient conservation, erosion control, soil degradation, and pollution remediation.

Variable charge minerals, such as kaolinite and goethite, are widely present in soil and environmental sciences, with their surface charges varying according to changes in ambient pH. Kaolinite’s primary chemical composition is aluminum silicate (Al_2_Si_2_O_5_(OH)_4_), with a layered crystal structure. Each layer consists of alternating silicon-oxygen tetrahedral and aluminum hydroxide octahedral sheets, leading to internal charge imbalances. The silicon-oxygen tetrahedral layers generally exhibit a stronger negative charge [1]. The layered structure endows it with unique physical and chemical properties, characterized by strong adsorption and ion-exchange capacities [2]. The chemical formula of goethite is FeO(OH), and it is an iron oxide mineral with mixed valence states. It is commonly recognized as the most prevalent crystalline iron oxide found in soils [3]. In goethite crystals, Fe(III) ions form octahedral coordination with oxygen and hydrogen. The octahedra share edges along the c-axis, creating a chain-like structure that arranges specifically in space to form the goethite crystal. This structure results in strong surface reactivity, high stability, and enhanced adsorption capacity [4].

Various methods are currently proposed to determine soil surface property parameters. Among methods for measuring surface potential, the zeta potential method is the most widely used and allows for direct measurement using instruments [5,6,7]. However, the zeta potential represents the potential at the shear plane during particle electrophoresis not the true surface potential. Numerous studies indicate that the measured zeta potential typically represents less than 20% of the actual surface potential [8,9,10]. The determination of surface charge is commonly conducted using potentiometric titration and ion exchange methods, but both methods have limitations when applied to variable charge colloids [11,12,13]. Factors such as pH, electrolyte type, concentration, and temperature are known to influence measurement results. Differences have also been observed in the surface charge values obtained by potentiometric titration and ion exchange methods [14]. The specific surface area is typically measured using gas adsorption or the methylene blue adsorption method, where an indicator adsorbate (with known molecular size) forms a monolayer on the soil particle surface. The product of the monolayer’s quantity and the area per molecule provides the specific surface area of soil particles [15,16,17]. However, this approach is suitable for minerals with significant external surfaces, and it may yield inaccurate results for expansive minerals with substantial internal surfaces, such as montmorillonite [18].

Two main methods are employed to determine surface charge density. The first method involves calculating surface charge density as the ratio of surface charge to specific surface area. However, since the surface state and properties of colloidal particles differ under gas-phase and liquid-phase conditions, the accuracy of using gas-phase specific surface area and liquid-phase surface charge for calculating surface charge density is questionable. The second method estimates surface charge density by measuring surface potential and applying the Gouy–Chapman theory. However, due to the lack of widely applicable methods for surface potential measurement, practical application faces significant challenges [19]. In complex soil colloid systems, surface property parameters are meaningful only when measured simultaneously under identical conditions.

The electric field generated by the surface charge of colloidal particles induces “non-classical polarization” in nearby ions, which is several hundred to several thousand times greater than classical polarizability [20,21,22]. This suggests a significant alteration in the quantum states of surface-adsorbed ions on colloidal particles. Using perturbation theory, solutions to the Schrödinger equation for the outer orbitals of atoms/ions on charged colloidal surfaces were successfully obtained. This provides rigorous quantum mechanical evidence that the orbitals of atoms/ions on charged soil colloidal surfaces undergo substantial changes. Consequently, the theory of asymmetric hybrid orbitals for interfacial atoms/ions is proposed [23,24]. The theory indicates that the non-classical polarization of surface-adsorbed ions on charged particles induces strong polarization effects with oxygen atoms on the particle surface, resulting in selective adsorption. Based on the non-classical polarization of ions, Li et al. proposed a method for the combined determination of the specific surface area and surface charge characteristics of charged particles through a series of ion-exchange experiments [25,26,27]. This method relies on the differences in adsorption capacity between two indicator ions, using a developed theoretical model to quantitatively evaluate each parameter. The Na/Ca indicator ion approach was successfully applied to various soil samples, including montmorillonite, TiO_2_, purple soil, and red soil [28,29,30,31]. However, Na⁺ ions do not exhibit polarization-induced covalent interactions at the particle surface, while Ca^2^⁺ ions exhibit strong Coulombic and polarization-induced covalent interactions. This leads to significant adsorption selectivity of Na/Ca on the sample surface, resulting in a much lower adsorption capacity for Na⁺ compared to Ca^2^⁺. Consequently, the measurement error for Na⁺ is difficult to control, requiring high experimental precision. To reduce the sensitivity of results to the adsorption selectivity of indicator ions, it is necessary to select ions with smaller differences in adsorption capacity. Both K⁺ and Ca^2^⁺ have outer electron configurations of 3s3p, resulting in polarization-induced covalent interactions [32], though Ca^2^⁺ has a stronger Coulombic effect than K⁺. Theoretically, the K/Ca ion system is more suitable than the Na/Ca system. This study aims to determine the surface properties of non-swelling variable charge minerals, such as kaolinite and goethite, using ion-selective electrode methods. This includes examining the effects of experimental conditions—such as pH, ion concentration ratios, and the method of adding solutions—on the measurement results. The optimal measurement approach within the K/Ca system is identified, providing a reference and laying the foundation for surface property measurements of other variable charge minerals. Regarding the methods for determining soil surface properties, the ion-selective electrode method combined with surface property analysis is currently the only approach capable of simultaneously measuring five surface property parameters within the same system. Other methods have significant limitations and shortcomings. The “soil” system developed in this study for comprehensive surface property analysis at the interface will overcome the current technical bottleneck in soil micro-property analysis, which often focuses on isolated aspects rather than providing a holistic understanding. This approach is expected to play a crucial role in advancing the field of soil science.

## 2. Materials and Methods

### 2.1. Experimental Materials and Pretreatment

In this study, variable charge minerals kaolinite and goethite (purchased from Shanghai Haohong Biopharmaceutical Technology Co., Ltd., Shanghai, China) are used as experimental materials. The sample used is kaolinite, with a chemical composition primarily consisting of silicon, aluminum, and oxygen, and its molecular formula is Al_2_Si_2_O_5_(OH)_4_. The sample appears white. Another sample, goethite, has a chemical composition mainly of iron and oxygen, with the molecular formula FeO(OH), and it appears yellow. The preparation of H⁺-saturated samples is shown as follows. A 500 g sample of kaolinite is placed in a 5 L beaker, and 2.5 L of a 0.1 mol/L HCl (Chuan Dong Chemical Co., Ltd., Nan’an District, Chongqing, China) solution is added at a soil-to-water ratio of 1:5. The mixture is stirred at 500 r/min for 24 h using a constant-temperature stirrer (Zhi Mei Technology Co., Ltd., Guangdong, China) (24 °C). After stirring, the suspension is transferred to centrifuge tubes and centrifuged at 6500 r/min for 10 min, and the supernatant is removed (Shanghai Luxiang Centrifuge Instrument Co., Ltd., Shanghai, China) (24 °C). The sample is then fully dispersed and returned to the beaker, where 2.5 L of a 0.1 mol/L HCl solution is added. This process is repeated four times under the same stirring conditions. Subsequently, the HCl solution is replaced with pure water, repeating the procedure twice more. After the final centrifugation, the supernatant is discarded, and the sample is spread evenly on a drying tray and dried at 75 °C for 48 h (Shanghai Qixin Scientific Instruments Co., Ltd., Shanghai, China). The dried sample is then ground to pass through a 0.25 mm sieve, homogenized, and stored in a sealed plastic bag. The preparation of the H⁺-saturated goethite sample follows the same procedure, with adjustments for its higher adhesiveness: the stirring speed is set to 800 r/min, and centrifugation is performed at 7500 r/min for 10 min.

### 2.2. Determination of Mineral Surface Properties

Preparation of Standard Solutions: In this method, ion activity is measured in mixed electrolyte solutions. According to the research by Tang Lingling et al., other ions present in mixed electrolyte solutions interfere with electrode measurements to some extent. Therefore, a mixed solution with equal ionic concentrations is used as the calibration standard [33]. The standard solutions used in this study have concentrations of 10^−5^, 10^−4^, 10^−3^, 10^−2^, and 10^−1^ mol L^−1^, respectively (Chuan Dong Chemical Co., Ltd., Nan’an District, Chongqing, China).

Calibration of Ion-Selective Composite Electrodes: A mixed standard solution is added to a beaker along with a magnetic stir bar. Prior to calibration, the electrode stability is tested using a 10^−3^ mol L^−1^ standard solution (Shanghai Leici Instruments Co., Ltd., Shanghai, China). The electrode is considered stable when the potential difference between two consecutive measurements is within ±1 mV. The sensing ends of the two ion-selective composite electrodes are then simultaneously immersed in the standard solution, and calibration is performed sequentially from lower to higher concentrations. The procedure involves stirring for 1 min at a speed of 300 r/min, followed by a 2 min resting period. Electrode potentials are recorded at 1 min 50 s, 2 min, and 2 min 10 s during the resting period, and the average of these three readings is taken as the final measured value [33]. Based on the potential of the sample solution, three concentrations are selected from the five concentration gradients of the standard solutions for calibration points, ensuring that the range of calibration potentials covers the potential range of the sample solution. Finally, a standard curve is plotted using the negative logarithm of the ionic activity (-lga) against the measured potential values (mV).

pH Meter Calibration and Standardization: Following the pH meter’s instructions, the pH meter is inserted into a standard buffer solution (pH = 6.86) for measurement. Once the reading stabilizes (after 1 min), the mV value is recorded. After rinsing the pH meter, measurements are taken sequentially in standard buffer solutions of pH = 9.18 and pH = 4.01 following the same procedure (Shanghai Leici Instruments Co., Ltd., Shanghai, China). A graph is plotted with pH on the *x*-axis and mV on the *y*-axis, and a linear fit is applied to obtain the standard curve.

Sample Preparation: Two electrolyte addition methods are employed in this experiment. ① Both KOH and Ca(OH)_2_ (Chuan Dong Chemical Co., Ltd., Nan’an District, Chongqing, China) alkaline solutions are added simultaneously. A beaker containing the sample is placed on a magnetic stirrer, and a KOH + Ca(OH)_2_ mixed solution is added. The mixture is stirred for 4 h, maintaining a speed that ensures no sediment at the bottom. The pH is measured and then adjusted to the target equilibrium pH using 0.5 mol L−^1^ CH_3_COOH. Stirring continues for an additional hour, after which the stabilized equilibrium pH is recorded. ② A KOH solution is added first, followed by a CaCl_2_ solution. The sample beaker is placed on a magnetic stirrer, and the KOH solution is introduced. The mixture is stirred for 3 h, and the pH is measured. The CaCl_2_ solution is then added, followed by 2 h of stirring. The pH is adjusted to the target equilibrium value using 0.5 mol L−^1^ CH_3_COOH, with stirring continuing for another hour before measuring the stabilized equilibrium pH.

Each method of solution addition presents distinct advantages and disadvantages.

KOH + Ca(OH)_2_ system: The primary advantage of this system lies in its use of two alkaline solutions, enabling effective neutralization of exchanged H⁺ ions. This facilitates a faster adsorption equilibrium of K⁺ and Ca^2^⁺, resulting in a shorter equilibrium time. However, this approach requires a mixed suspension, limiting the feasible concentration, and demands immediate, uniform mixing for accurate results. KOH + CaCl_2_ system: This method benefits from sequential addition, which avoids forming a suspension, making the process simpler and more efficient. Nevertheless, since only KOH provides OH⁻ for H⁺ neutralization, a larger amount of KOH is needed, leading to an excessive K⁺ concentration in the system. This results in higher equilibrium concentrations and greater measurement errors. Additionally, once K⁺ is adsorbed onto the mineral surface, the replacement of K⁺ by Ca^2^⁺ requires higher activation energy, thereby extending the equilibrium time.

Measurement of the Test Solution: After equilibrium is reached, the electrode potentials of K⁺ and Ca^2^⁺ in the test solution are measured using the same procedure as employed during electrode calibration.

### 2.3. Study on the Effect of pH Conditions on Surface Property Measurements

When ion-selective electrode methods are used to measure surface property parameters, two critical pH values are involved. The first pH value is the initial pH observed after adding the electrolyte solution to the sample and reaching equilibrium through stirring. The second pH value is the equilibrium pH at which the ion-selective electrode measurement is performed after pH adjustment of the test solution. By setting different initial and equilibrium pH conditions, the impact of pH on the measurement of surface property parameters is explored, and the optimal pH range is determined. For kaolinite, the initial pH is set at 7.5, 8.5, and 9.0, with an equilibrium pH range of 7.5 to 8.0 [33]. For goethite, the initial pH values are set at 8.0 and 9.0, with corresponding equilibrium pH values of 8.0 and 9.0.

### 2.4. Study on the Effect of Ion Concentration Ratios on Surface Property Measurements

Differences in adsorption energy between two indicator ions in the electrolyte solution can cause uneven distribution within the interlayer structure, leading to adsorption imbalance. Based on these differences, two mixed electrolyte systems—KOH + Ca(OH)_2_ and KOH + CaCl_2_—are used to adjust ion concentration ratios, facilitating adsorption equilibrium between the ions and determining the optimal mixed system and ion concentration ratio. In the KOH + Ca(OH)_2_ system, the K⁺ to Ca^2^⁺ concentration ratios (*c*_K_:*c*_Ca_) for kaolinite are set at 1:1, 1.5:1, and 2:1, while for goethite, they are set at 4:1 and 5:1. In the KOH + CaCl_2_ system, the K⁺ to Ca^2^⁺ ratios for kaolinite are adjusted to 7:1, 8:1, and 9:1, and for goethite, they are set at 18:1 and 20:1.

### 2.5. Experimental Principle and Surface Property Calculation Formula

The principle of multi-parameter surface property analysis involves using two ions with different interfacial adsorption energies (e.g., K⁺ and Ca^2^⁺) as indicator ions. The activity or concentration of these ions in the equilibrium suspension is measured during adsorption equilibrium experiments. These measurements are then used to characterize surface property parameters through relevant calculation formulas. In this study, K⁺ and Ca^2^⁺ are employed as indicator ions. Their activities in the equilibrium suspension are measured using ion-selective electrodes, and their concentrations are calculated using an iterative algorithm.

#### 2.5.1. Iterative Conversion of Ion Activity to Concentration

The iterative algorithm proposed by Liu is used to convert ion activity into concentration [28]. Taking the K/Ca system as an example, the relationship between the electrode potential of the ion-selective electrode and ion activity follows the Nernst equation.

(1) $$E_{K}=E_{K}^{0}+\frac{RT}{F}\ln a_{K}^{0}$$

(2) $$E_{\mathrm{Ca}}=E_{\mathrm{Ca}}^{0}+\frac{RT}{2F}\ln a_{\mathrm{Ca}}^{0}$$

In the equation, *E*_K_ and *E*_Ca_ (V) represent the electrode potentials of the K⁺ and Ca^2^⁺ ion-selective electrodes, respectively, while $E_{K}^{0}$ and $E_{\mathrm{Ca}}^{0}$ denote their standard electrode potentials. $a_{K}^{0}$ and $a_{\mathrm{Ca}}^{0}$ (mol/L) are the activities of K^+^ and Ca^2+^, respectively. *R* (J/(K·mol)) is the gas constant, *T* (K) is the temperature in Kelvin, and *F* (C/mol) is the Faraday constant.

Setting $a_{K}^{0}$ and $a_{\mathrm{Ca}}^{0}$ as the initial concentrations of K⁺ and Ca^2^⁺, denoted as *c*_K_(0) and *c*_Ca_(0), the ionic strength for the first iteration is calculated as

(3) $$I(1)=\frac{1}{2}\sum c_{i}(0){Z_{i}}^{2}=c_{K}(0)+3c_{\mathrm{Ca}}(0)$$

In the equation, *I*(1) denotes the ionic strength in the first iteration, and *Z* represents the ionic valence.

The activity coefficients of K⁺ and Ca^2^⁺ in the first iteration are expressed as follows:

(4) $$\mathrm{lg}\gamma_{K}(1)=-0.5102\left(\frac{\sqrt{I\left(1\right)}}{1+\sqrt{I\left(1\right)}}-0.3I\left(1\right)\right)$$

(5) $$\mathrm{Ig}\gamma_{\mathrm{Ca}}\left(1\right)=-2.0408\left(\frac{\sqrt{I\left(1\right)}}{1+\sqrt{I\left(1\right)}}-0.3I\left(1\right)\right)$$

Thus, the concentrations of K⁺ and Ca^2^⁺ in the first iteration are calculated as follows:

(6) $$c_{K}\left(1\right)=\frac{a_{K}^{0}}{\gamma_{K}\left(1\right)}$$

(7) $$c_{\mathrm{Ca}}\left(1\right)=\frac{a_{\mathrm{Ca}}^{0}}{\gamma_{\mathrm{Ca}}\left(1\right)}$$

Similarly, the ionic strength in the second iteration is calculated as

(8) $$I(2)=\frac{1}{2}\sum c_{i}(1){Z_{i}}^{2}=c_{K}(1)+3c_{\mathrm{Ca}}(1)$$

The corresponding activity coefficients are given as follows:

(9) $$\mathrm{lg}\gamma_{K}(2)=-0.5102\left(\frac{\sqrt{I(2)}}{1+\sqrt{I(2)}}-0.3I(2)\right)$$

(10) $$\mathrm{lg}\gamma_{\mathrm{Ca}}(2)=-2.0408\left(\frac{\sqrt{I(2)}}{1+\sqrt{I(2)}}-0.3I(2)\right)$$

The concentrations of K^+^ and Ca^2+^ in the second iteration are as follows:

(11) $$c_{K}(2)=\frac{a_{K}^{0}}{\gamma_{K}(2)}$$

(12) $$c_{\mathrm{Ca}}(2)=\frac{a_{\mathrm{Ca}}^{0}}{\gamma_{\mathrm{Ca}}(2)}$$

The concentrations of K^+^ and Ca^2+^ after the *i*-th iteration are determined as follows:

(13) $$c_{K}\left(i\right)=\frac{a_{K}^{0}}{\gamma_{K}\left(i\right)}$$

(14) $$c_{\mathrm{Ca}}\left(i\right)=\frac{a_{\mathrm{Ca}}^{0}}{\gamma_{\mathrm{Ca}}\left(i\right)}$$

The iteration is terminated when the condition [*I*(*k* + 1) − *I*(*k*)]/*I*(*k* + 1) < 0.001 is met at *i* = *k* + 1. At this point, *c*_K_(*i*) and *c*_Ca_(*i*) are considered the equilibrium concentrations of K^+^ and Ca^2+^, respectively, after the iteration.

#### 2.5.2. Calculation Formulas for Surface Properties

The concentration changes of indicator ions before and after the interfacial reaction are measured using ion-selective electrodes. Based on these measurements, a series of equations is employed to calculate surface properties of the soil, including surface potential, charge density, total charge, specific surface area, and electric field strength [20,34].

(1) Surface Potential

(15) $$\varphi_{0}=\frac{2RT}{\left(2\beta_{Ca}-\beta_{K}\right)F}\ln\frac{a_{Ca}^{0}N_{K}}{a_{K}^{0}N_{\mathrm{Ca}}}$$

In this equation, *φ*_0_ (V) represents the surface potential. *β*_K_ and *β*_Ca_ are the relative charge coefficients for K^+^ and Ca^2+^ in the K/Ca exchange system, with values of *β*_K_ = 0.984 and *β*_Ca_ = 1.016 [20]. N_K_ and N_Ca_ (mol/g) denote the adsorption amounts of K^+^ and Ca^2+^ in the diffuse layer at equilibrium.

(16) $$N_{K}=\left[\left(c_{K}-c_{K}^{0}\right)V/W\right]\times 10^{5}$$

(17) $$N_{\mathrm{Ca}}=\left[\left(c_{\mathrm{Ca}}-c_{\mathrm{Ca}}^{0}\right)V/W\right]\times 10^{5}$$

In this equation, *c*_K_ and *c*_Ca_ (mol/L) represent the initial concentrations of K^+^ and Ca^2+^, respectively, *V* (L) denotes the volume of the solution, and *W* (g) indicates the mass of the soil sample. Once the adsorption amounts of K^+^ and Ca^2+^ are obtained, the total ion adsorption capacity of the soil can be expressed as

(18) $$N=N_{K}+2N_{\mathrm{Ca}}$$

(2) Surface Charge Density

(19) $$\sigma_{0}=\mathrm{sgn}\left(\varphi_{0}\right)\sqrt{\frac{\varepsilon PT}{2\pi }\left(a_{K}^{0}\exp\left(-\frac{\beta_{K}F\varphi_{0}}{RT}\right)+a_{\mathrm{Ca}}^{0}\exp\left(-\frac{2\varphi_{\mathrm{Ca}}F\varphi_{0}}{RT}\right)\right)}$$

In this equation, *σ*_0_ (C/dm^2^) represents the surface charge density, and ε denotes the dielectric constant of the medium, which is 8.9 × 10^−10^ C^2^ J^−1^ dm^−1^ in water.

(3) Surface Electric Field Intensity

(20) $$E_{0}=\frac{4\pi }{\varepsilon }\sigma_{0}$$

(4) Surface Charge Quantity

(21) $$T_{C_{0}}=(N_{K}+2N_{\mathrm{Ca}})\times 10^{5}$$

In this equation, *T*_C0_ (cmol/kg) denotes the surface charge quantity of the soil sample.

(5) Specific Surface Area

(22) $$S=\frac{\left(N_{K}+2N_{\mathrm{Ca}}\right).F}{\sigma_{0}}$$

In this equation, *S* (m^2^/g) represents the specific surface area.

(6) Ion Adsorption Ratio

(23) $$n_{K}=\frac{c_{K}^{0}}{c_{K}}$$

(24) $$n_{\mathrm{Ca}}=\frac{c_{\mathrm{Ca}}^{0}}{c_{\mathrm{Ca}}}$$

In this equation, *n*_K_ and *n*_Ca_ denote the ion adsorption ratios, while $c_{K}^{0}$ and $c_{\mathrm{Ca}}^{0}$ (mol/L) represent the equilibrium concentrations of K^+^ and Ca^2+^, respectively.

The above Equations (1)–(24) are used to calculate surface property parameters based on the measured activity of the indicator ions. From a computational perspective, the calculated value of specific surface area (*S*) is correlated with surface charge density (*σ*_0_), surface potential (*φ*_0_), and surface electric field intensity (*E*_0_). Therefore, specific surface area (*S*) serves as a critical indicator for assessing the reliability of the measurement results.

#### 2.5.3. Experimental Methods and Technical Roadmap

The experimental methods and technical roadmap in this study are showed as follows. 


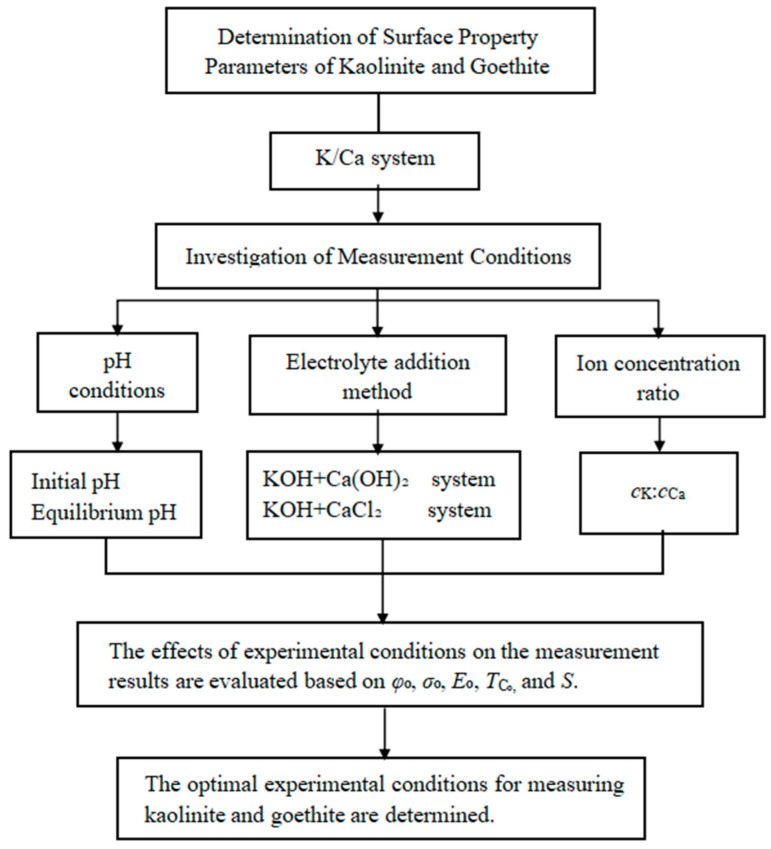


## 3. Results

### 3.1. The Influence of pH Conditions on the Measurement of Surface Properties of Kaolinite and Goethite

A mixed electrolyte solution with a concentration ratio of 1:1 (2.00 mmol/L KOH + 2.00 mmol/L Ca(OH)_2_) is selected, with an initial pH of 7.5, 8.5, and 9.0 and an equilibrium pH of 7.8 ± 0.2. The results are shown in Table 1.

**Figure 1:**
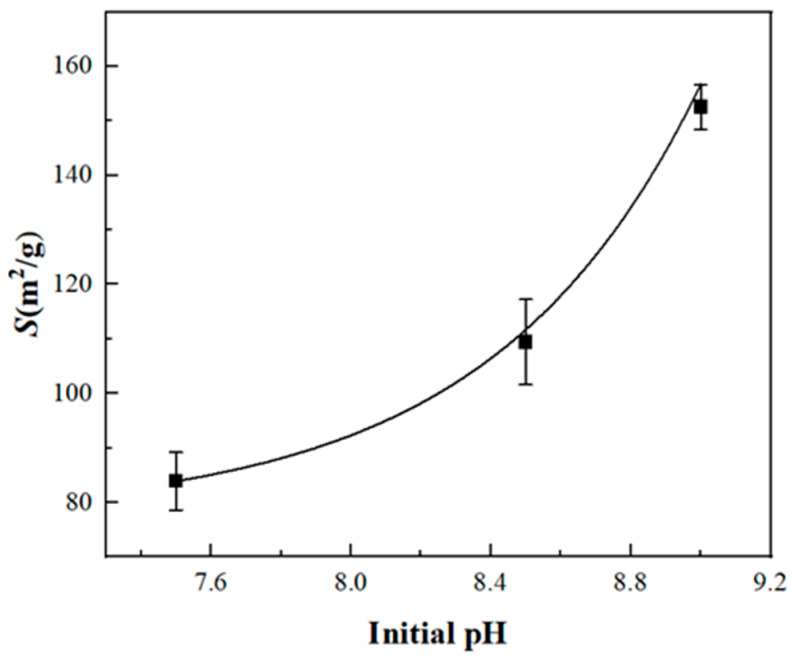
The relationship between the measured specific surface area and the initial pH conditions of kaolinite.

**Figure A1:**
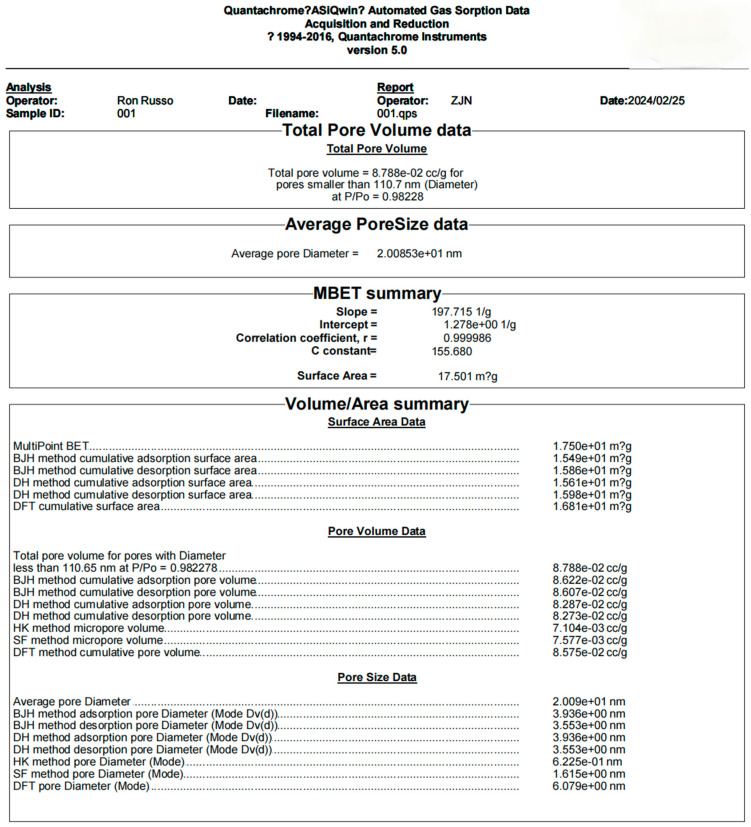
The specific surface area of the saturated sample of H^+^-kaolinite measured by the BET method.

Table 1 and Figure 1 demonstrate that in the determination of kaolinite within the K/Ca system at a *c*_K_: *c*_Ca_ ratio of 1:1, the surface potential, surface charge density, and surface electric field intensity increase progressively with rising initial pH, while surface charge quantity and specific surface area decrease correspondingly. Under initial pH conditions of 7.5–9.0, the measured specific surface area ranges from 83.3 to 152.5 m^2^/g, significantly exceeding the BET measurement result of 17.501 m^2^/g (Appendix A, Figure A1). At initial pH values of 7.5–9.0, the measured surface charge quantity (*T*_C0_) varies between 4.28–3.90 cmol/kg, with N_K_ ranging from 0.91 to 0.77 cmol/kg and N_Ca_ from 1.70 to 1.56 cmol/kg. Additionally, *n*_K_ increases from 0.547 to 0.724, while *n*_Ca_ rises from 0.157 to 0.443.

The results indicate that as the initial pH increases, the ion adsorption decreases, particularly for K^+^, leading to a significant reduction in K^+^ adsorption. This creates an imbalance in the adsorption states of K^+^ and Ca^2+^. The increased n_K_ and n_Ca_ values suggest that more K^+^ and Ca^2+^ remain unadsorbed at binding sites, existing in a free state. This reflects an uneven adsorption between the two indicator ions, ultimately resulting in inaccurate measurements of surface properties.

The specific surface area is determined by the ion adsorption capacity and surface charge density. Under aqueous conditions, as the initial pH increases, a greater volume of alkaline electrolyte solution is required, which further disperses the interstitial spaces within the sample structure, leading to an increase in exposed specific surface area. This results in a higher number of negative charges and increased adsorption of positively charged ions. However, the stronger repulsive forces make it difficult for ions to effectively adsorb onto binding sites, preventing the two indicator cations from reaching adsorption equilibrium. Additionally, the adsorption capacity of a saturated sample of a given mass has an upper limit. When the concentration of the added alkaline electrolyte solution remains constant, a higher volume leads to more OH⁻ as well as K⁺ and Ca^2^⁺ indicator ions. This results in adsorption amounts being significantly lower than the amount added, which complicates ion-selective electrode measurements and increases experimental error.

Goethite, as a variable charge mineral, also exhibits surface charge responses to changes in external pH. Therefore, when using ion-selective electrodes to determine the surface property parameters of goethite, two critical pH values are involved: the initial pH and the equilibrium pH. A mixed electrolyte solution with a *c*_K_:*c*_Ca_ ratio of 1:1 (2.00 mmol/L KOH + 2.00 mmol/L Ca(OH)_2_) is used as the initial concentration ratio. The initial pH is set at 8.0 and 9.0, while the equilibrium pH is also maintained at 8.0 and 9.0 for the measurement of surface properties. The results are presented in Table 2 and Table 3.

Table 2 indicates that ion adsorption varies under initial pH conditions of 8.0 and 9.0. When both the initial and equilibrium pH are 8.0, the average adsorption of K⁺ is 1.01 cmol/kg, while that of Ca^2^⁺ is 3.00 cmol/kg, with *n*_K_ at 0.829 and *n*_Ca_ at 0.496. The total ion adsorption is low, failing to effectively occupy all binding sites. At an initial pH of 9.0 and an equilibrium pH of 8.0, the average adsorption of K⁺ decreases to 0.89 cmol/kg, while that of Ca^2^⁺ increases to 3.39 cmol/kg, leading to a higher total ion adsorption. The values of *n*_K_ at 0.880 and *n*_Ca_ at 0.545 indicate an increase in the ion adsorption ratio. When both the initial and equilibrium pH are 9.0, the adsorption of K⁺ and Ca^2^⁺ is significantly higher compared to the other two pH conditions, while the ion adsorption ratio decreases. This suggests that K⁺ and Ca^2^⁺ occupy more binding sites, resulting in fewer free indicator ions within the system.

**Figure A2:**
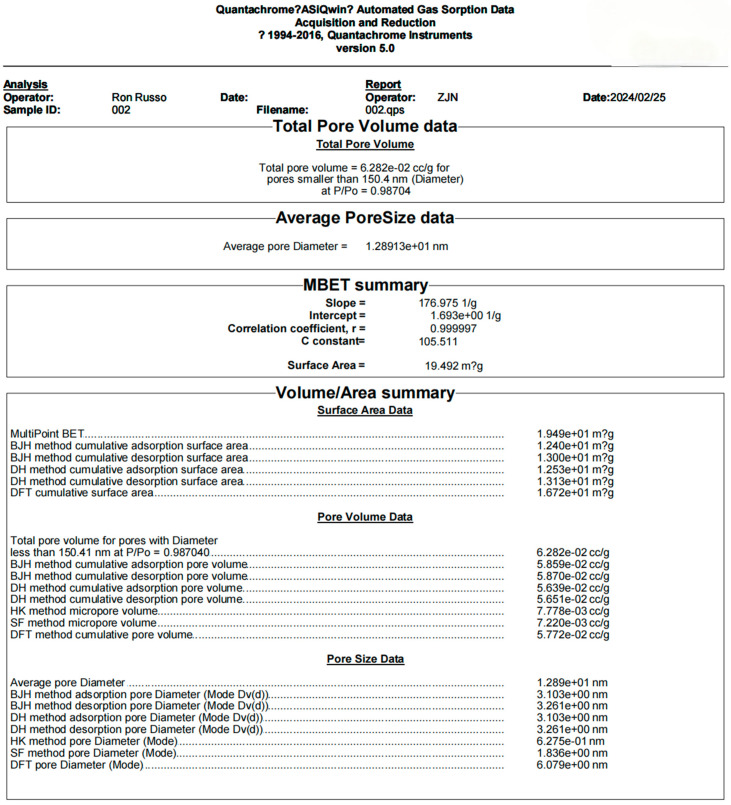
Specific surface area of H^+^-goethite saturated sample measured by BET method.

Table 3 shows that under initial and equilibrium pH conditions of 8.0 to 9.0, the measured specific surface area ranges from 82.8 to 120.3 m^2^/g, which is entirely unreasonable and significantly exceeds the BET measurement result of 19.492 m^2^/g (Appendix A, Figure A2). When both the initial and equilibrium pH are 8.0 and 9.0, the specific surface areas measured are 120.3 m^2^/g and 142.3 m^2^/g, respectively. However, when the initial pH is 9.0 and the equilibrium pH is 8.0, the specific surface area decreases to 82.8 m^2^/g. It is concluded that under all three pH conditions, the adsorption of K⁺ and Ca^2^⁺ is imbalanced, failing to effectively occupy the binding sites, which leads to the inaccurate determination of surface property parameters.

The findings suggest two key considerations for controlling initial pH conditions. First, the initial pH should be maintained above 7.0 to ensure complete displacement and consumption of H⁺ from the H⁺-saturated sample, thereby reducing competition with K⁺ and Ca^2^⁺ for adsorption at binding sites. Second, the initial pH affects the adsorption capacity of the indicator cations and the ion adsorption ratio, making it difficult to achieve adsorption equilibrium.

Establishing the equilibrium pH conditions requires meeting two criteria. First, the equilibrium pH must exceed the pH necessary for the cation exchange capacity (CEC) of the sample to ensure sufficient adsorption of indicator cations. Second, the difference between the equilibrium pH and the initial pH should be within the range of 0.5–1.0 pH units, enabling effective occupation of binding sites by the indicator cations.

### 3.2. Impact of Ion Concentration Ratio on the Determination of Surface Property Parameters of Kaolinite and Goethite

In this experiment, K⁺ and Ca^2^⁺ are used as indicator ions for adsorption, and the surface property parameters are determined based on the differences in their adsorption energies. Within the K/Ca system, K⁺ is considered a weakly adsorbing ion and does not easily reach adsorption equilibrium with Ca^2^⁺.

To address this, two electrolyte systems are employed: a KOH + Ca(OH)_2_ mixed system and a KOH + CaCl_2_ mixed system. The ion concentration ratio is adjusted to influence the adsorption ratio and equilibrium rate of K⁺ and Ca^2^⁺. This approach aims to identify the optimal ion concentration ratio and differences in adsorption behavior across the mixed systems.

In the KOH + Ca(OH)_2_ mixed system for kaolinite, K⁺ is a weaker adsorbing ion at a concentration ratio of *c*_K_:*c*_Ca_ = 1:1, with Ca^2^⁺ occupying most of the binding sites. Increasing the initial concentration of K⁺ promotes faster attainment of adsorption equilibrium between K⁺ and Ca^2^⁺. Therefore, a mixed electrolyte solution with an initial concentration of 2.00 mmol/L KOH and 2.00 mmol/L Ca(OH)_2_ is used. The experiments are conducted at initial concentration ratios of *c*_K_:*c*_Ca_ = 1:1 (2.00 mmol/L KOH + 2.00 mmol/L Ca(OH)_2_), *c*_K_:*c*_Ca_ = 1.5:1 (3.00 mmol/L KOH + 2.00 mmol/L Ca(OH)_2_), and *c*_K_:*c*_Ca_ = 2:1 (3.00 mmol/L KOH + 1.50 mmol/L Ca(OH)_2_) under initial pH conditions of 7.8 ± 0.3 and equilibrium pH conditions of 7.8 ± 0.2. The results are presented in Table 4 and Table 5.

Table 4 shows that as the initial concentration ratio of K⁺ increases, its adsorption also rises, while the adsorption of Ca^2^⁺ decreases correspondingly. At *c*_K_:*c*_Ca_ ratios ranging from 1:1 to 2:1, the average adsorption of K⁺ increases from 0.905 cmol/kg to 1.42 cmol/kg, while the average adsorption of Ca^2^⁺ decreases from 1.69 cmol/kg to 1.54 cmol/kg. This indicates that increasing the K⁺ concentration ratio effectively enhances competitive adsorption, occupying binding sites and promoting adsorption equilibrium with Ca^2^⁺.

**Figure 2:**
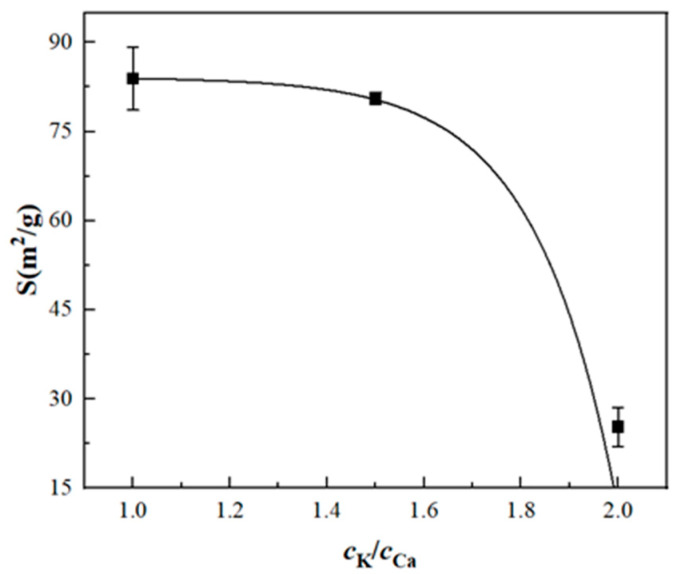
The relationship between the specific surface area and the ion concentration ratio of kaolinite.

Table 5 and Figure 2 indicate that the ion concentration ratio directly affects the determination of surface property parameters. In the measurement of kaolinite’s surface properties, increasing the concentration ratio of K⁺ leads to an increase in surface potential, surface charge density, surface electric field intensity, and surface charge quantity, while the specific surface area shows a declining trend. At a *c*_K_:*c*_Ca_ ratio of 2:1, the specific surface area is 83.9 m^2^/g, exceeding the BET measurement, as Ca^2^⁺ occupies too many binding sites, resulting in relatively low K⁺ adsorption. However, when the *c*_K_:*c*_Ca_ ratio is adjusted to 2:1, the specific surface area decreases to 25.3 m^2^/g, aligning closely with the BET result. This demonstrates that a *c*_K_:*c*_Ca_ ratio of 2:1 enables K⁺ to effectively occupy binding sites while preventing excessive adsorption of Ca^2^⁺, facilitating a more effective adsorption equilibrium between the two indicator ions.

The results from the KOH + Ca(OH)_2_ mixed system indicate that when K⁺ and Ca^2^⁺ undergo competitive adsorption, the relatively lower adsorption energy of K⁺ often leads to an imbalance between the two indicator ions.

To address this, in the KOH + CaCl_2_ mixed system, an alkaline solution containing the weakly adsorbing ion (K⁺) is added first, allowing K⁺ to initiate the adsorption–displacement reaction. Once the system reaches the desired initial pH, a salt solution containing the strongly adsorbing ion (Ca^2^⁺) is introduced, enabling Ca^2^⁺ to undergo adsorption–displacement. This approach ultimately achieves adsorption equilibrium.

Therefore, an electrolyte solution with a lower concentration of KOH and a higher concentration of CaCl_2_ is used, with the initial pH set at 8.2 ± 0.2 and the equilibrium pH at 7.8 ± 0.2. Surface properties are measured at ion concentration ratios of *c*_K_:*c*_Ca_ = 7:1, 8:1, and 9:1. The results are presented in Table 6 and Table 7.

Table 6 shows that in the KOH + CaCl_2_ mixed system, the adsorption ratio of K⁺ and Ca^2^⁺ is influenced by the ion concentration ratio. At *c*_K_:*c*_Ca_ ratios ranging from 7:1 to 9:1, K⁺ adsorption increases from 2.35 cmol/kg to 2.51 cmol/kg, while Ca^2^⁺ adsorption decreases from 0.87 cmol/kg to 0.72 cmol/kg. This indicates that K⁺ effectively occupies the binding sites, facilitating the attainment of adsorption equilibrium between the two indicator ions.

**Figure 3:**
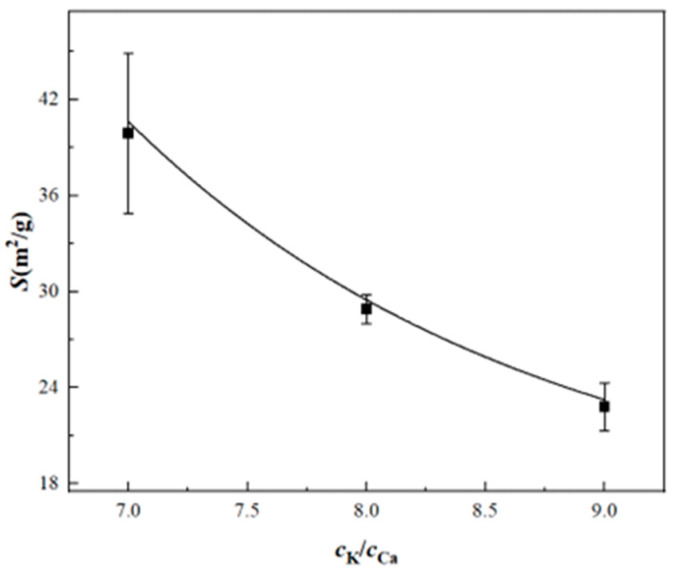
The relationship between the specific surface area and the ion concentration ratio of kaolinite.

Table 7 and Figure 3 indicate that the ion concentration ratio in the KOH + CaCl_2_ mixed system affects the determination of surface property parameters. At a *c*_K_:*c*_Ca_ ratio of 7:1, the specific surface area is 39.9 m^2^/g, significantly exceeding the BET measurement. When the ratio is adjusted to 9:1, the specific surface area decreases to 22.8 m^2^/g, closely aligning with the BET result. This suggests that at a *c*_K_:*c*_Ca_ ratio of 9:1, K⁺ and Ca^2^⁺ achieve adsorption equilibrium more effectively under the same conditions, resulting in higher reliability of the measured surface property parameters.

The results from both mixed systems demonstrate that the surface property parameters of kaolinite can be effectively measured under specific conditions. In the KOH + Ca(OH)_2_ system, the optimal ion concentration ratio is *c*_K_:*c*_Ca_ = 2:1, while in the KOH + CaCl_2_ system, the optimal ratio is *c*_K_:*c*_Ca_ = 9:1, indicating a significant difference between the two systems.

In the KOH + Ca(OH)_2_ system, K⁺ and Ca^2^⁺ adsorb simultaneously, and differences in their adsorption capacity reflect variations in their outer electron structures [24,32] (Based on the outer electron structure, K⁺ has a stronger polarization ability than Ca^2^⁺ [32], but Ca^2^⁺, being divalent, exhibits stronger Coulombic attraction. This combination results in relatively similar adsorption capacities, making it necessary to limit the concentration ratio.

In the KOH + CaCl_2_ system, KOH is added first to facilitate K⁺ adsorption and achieve the desired pH before CaCl_2_ is introduced for Ca^2^⁺ adsorption. The OH⁻ required to reach the initial pH is solely provided by KOH, allowing an increased K⁺ concentration ratio to prevent excessive occupation of binding sites by Ca^2^⁺. This approach enables a more effective adsorption equilibrium between the two indicator ions.

The results for goethite in the KOH + Ca(OH)_2_ mixed system at a *c*_K_:*c*_Ca_ ratio of 1:1 show that K⁺, as the weaker adsorbing ion, struggles to achieve adsorption equilibrium with Ca^2^⁺. Increasing the initial concentration of K⁺ facilitates a faster attainment of adsorption equilibrium between the two indicator ions. Therefore, the experiments are conducted under initial pH conditions of 9.0 and equilibrium pH conditions of 8.0, with ion concentration ratios set at *c*_K_:*c*_Ca_ = 4:1 (4.00 mmol/L KOH + 1.00 mmol/L Ca(OH)_2_) and *c*_K_:*c*_Ca_ = 5:1 (5.00 mmol/L KOH + 1.00 mmol/L Ca(OH)_2_). The results are presented in Table 8 and Table 9.

Table 8 shows that the ion concentration ratio in the KOH + Ca(OH)_2_ mixed system affects ion adsorption on goethite. At a *c*_K_:*c*_Ca_ ratio of 4:1, the average adsorption of K⁺ is 1.41 cmol/kg while that of Ca^2^⁺ is 2.21 cmol/kg. When the ratio increases to 5:1, the average adsorption of K⁺ rises to 1.90 cmol/kg, while Ca^2^⁺ decreases to 2.03 cmol/kg. Similar to the results observed with kaolinite, increasing the concentration ratio of K⁺ effectively enhances its competitive adsorption capacity, facilitating equilibrium with Ca^2^⁺.

Table 9 shows that at a *c*_K_:*c*_Ca_ ratio of 4:1, K⁺ and Ca^2^⁺ effectively reach adsorption equilibrium, resulting in the higher accuracy of the measured surface property parameters. The specific surface areas measured at *c*_K_:*c*_Ca_ ratios of 4:1 and 5:1 are 21.7 m^2^/g and 26.91 m^2^/g, respectively, with the 4:1 ratio more closely aligning with the BET value for goethite used in this experiment. At a *c*_K_:*c*_Ca_ ratio of 4:1, K⁺ and Ca^2^⁺ achieve a better adsorption equilibrium under the same conditions, leading to more reliable surface property measurements. In contrast, at a *c*_K_:*c*_Ca_ ratio of 5:1, the higher K⁺ concentration results in greater adsorption, occupying more binding sites and preventing effective equilibrium between the two ions.

The results from the KOH + Ca(OH)_2_ mixed system indicate that K⁺ struggles to reach adsorption equilibrium with Ca^2^⁺ at low initial concentration ratios, partly due to the suspension state of Ca(OH)_2_, which introduces experimental errors. To address this issue, a KOH + CaCl_2_ mixed system is used, where K⁺ is allowed to undergo initial adsorption, followed by the introduction of Ca^2^⁺ for competitive adsorption after a set period. This approach ultimately enables both ions to achieve adsorption equilibrium.

Therefore, a mixed electrolyte solution with a lower concentration of KOH and a higher concentration of CaCl_2_ is used. The initial pH is set at 8.2 ± 0.2, and the equilibrium pH is set at 7.8 ± 0.2. Measurements are conducted at concentration ratios of *c*_K_:*c*_Ca_ = 18:1 and 20:1. The results are presented in Table 10 and Table 11.

Table 10 shows that in the KOH + CaCl_2_ mixed system, the adsorption ratio of K⁺ and Ca^2^⁺ is influenced by the ion concentration ratio. At *c*_K_:*c*_Ca_ ratios of 4:1 and 5:1, the adsorption amounts of K⁺ and Ca^2^⁺ are similar, with nearly identical adsorption ratios (*n*_K_ and *n*_Ca_). As the concentration ratio of K⁺ increases, K⁺ adsorption rises while Ca^2^⁺ adsorption decreases, facilitating faster attainment of adsorption equilibrium between the two ions.

Table 11 indicates that at a *c*_K_:*c*_Ca_ ratio of 20:1, K⁺ and Ca^2^⁺ effectively achieve adsorption equilibrium. The measured specific surface areas at *c*_K_:*c*_Ca_ ratios of 18:1 and 20:1 are 27.2 m^2^/g and 21.5 m^2^/g, respectively, with the 20:1 ratio being closer to the BET value for goethite. At this concentration ratio, K⁺ more effectively occupies binding sites, promoting equilibrium between the two ions. The optimal ion concentration ratio is *c*_K_:*c*_Ca_ = 4:1 in the KOH + Ca(OH)_2_ system and *c*_K_:*c*_Ca_ = 20:1 in the KOH + CaCl_2_ system, indicating a significant difference between the two systems, similar to the results observed with kaolinite.

## 4. Discussion

### 4.1. The Experimental Conditions for Kaolinite and Goethite Differ in the K/Ca Measurement System

Significant differences are observed in the measurement conditions for kaolinite and goethite within the K/Ca system, with distinct requirements in the KOH + Ca(OH)_2_ and KOH + CaCl_2_ mixed systems. The pH conditions and ion concentration ratios notably impact the determination of surface property parameters. Through analysis and discussion, optimal measurement conditions for each mixed system are identified, and the further analysis of measurement conditions for additional materials is conducted.

Optimal measurement conditions for kaolinite and goethite differ across mixed systems, with minimal variance in pH but significant differences in ion concentration ratios. As indicated in Table 1 and Table 2, the basic initial pH requirement is set between 7.0 and 10.0, ensuring complete neutralization of H⁺ on H⁺-saturated samples by OH⁻, thereby allowing the indicator cation to occupy binding sites. However, excessively high initial pH levels can increase measurement difficulty and experimental errors. For kaolinite, an optimal initial pH range of 7.5 to 8.5 is used, while for goethite, the range is slightly higher at 8.5 to 9.0; both ranges maintain mildly alkaline conditions.

The differences in the initial pH conditions for kaolinite and goethite in measurements are attributed to their mineral compositions, crystal structures, and surface chemical properties. Kaolinite has a layered structure formed by alternating silicon-oxygen tetrahedra and aluminum-oxygen octahedra, connected by hydrogen bonds between layers. This structure results in fewer surface active sites, with charge variation primarily dependent on edge hydroxyl groups [35,36]. These hydroxyl groups undergo protonation or deprotonation with pH changes, leading to the formation of either positive or negative charges. However, due to the high stability of kaolinite’s layered structure and its relatively low surface activity, it exhibits weak variable charge capacity [37,38].

In contrast, goethite’s crystal structure consists of octahedral chains of iron and oxygen, which provide abundant surface Fe-OH groups. These groups generate significant variable charge characteristics through proton adsorption or dissociation. The surface iron hydroxyl groups (Fe-OH) of goethite exhibit high chemical reactivity, capable of significantly releasing or absorbing protons as pH changes, leading to noticeable surface charge alterations [39,40].

Overall, kaolinite’s variable charge capacity is limited and primarily relies on the reactivity of edge hydroxyl groups, while goethite demonstrates significant variable charge properties due to the high reactivity of its surface iron hydroxyl groups under changing pH conditions. These differences collectively account for the variation in their initial pH requirements.

The equilibrium pH conditions for kaolinite and goethite in various systems are both in the range of 7.5 to 8.0, which are related to their respective points of zero charge (PZC) and cation exchange capacity (CEC). The equilibrium pH is set above the PZC of the minerals to ensure that their surfaces carry a negative charge. This allows the adsorption of indicator cations, facilitating the determination of surface properties. The CEC of kaolinite is also influenced by pH conditions; however, unlike goethite, kaolinite has a portion of permanent charge, making its CEC relatively stable. In contrast, the CEC of goethite is entirely dependent on pH, as it consists solely of variable charges.

The point of zero charge (pH PZC) of kaolinite typically ranges from 3.2 to 4.3 [41]. Under low pH conditions (acidic environment, pH < 3.2), the cation exchange capacity (CEC) of kaolinite is low, with variable charges primarily contributed by edge hydroxyl (OH⁻) groups. At a low pH, these edge hydroxyl groups undergo protonation (gain of H⁺), resulting in a positively charged surface that reduces the capacity for cation adsorption. When the pH conditions (pH = 3.2–4.3) are close to the pH PZC of kaolinite, surface charge fluctuations are minimal, and the CEC remains relatively low. This is mainly attributed to the limited active sites on the kaolinite surface, with charge exchange occurring only at edge hydroxyl groups.

In alkaline conditions (pH > 7.0), the edge hydroxyl groups of kaolinite gradually undergo deprotonation, creating a negatively charged surface that enhances cation adsorption, leading to a relative increase in CEC. However, due to the lower density of active sites on kaolinite compared to goethite, the increase in CEC under alkaline conditions is still limited. As a result, the equilibrium pH of kaolinite typically stabilizes around 7.5 to 8.0, where its surface is sufficiently active to effectively adsorb indicator cations.

The cation exchange capacity (CEC) of goethite is pH-dependent, with its point of zero charge (pH PZC) generally ranging from 7.0 to 9.0 [42,43]. Under low pH conditions (pH < 7.0), the Fe-OH groups on the goethite surface readily undergo protonation to form Fe-OH_2_⁺, resulting in a positively charged surface. This positive charge generates a strong repulsion toward cations, thereby hindering their adsorption and leading to a near-zero CEC. At neutral pH conditions (pH ≈ 7.0), the surface charge approaches the pH PZC, resulting in a weak cation exchange capacity as the surface maintains a balanced state of positive and negative charges. In high pH conditions (alkaline environment, pH > 7.0), the goethite surface becomes negatively charged, which enhances its ability to adsorb cations, resulting in an increased CEC. Consequently, the equilibrium pH of goethite is typically maintained between 7.5 and 8.0, ensuring effective adsorption of indicator cations.

In summary, for different measurement materials, the initial pH should be set between 7.0 and 11.0 to establish an equilibrium system that maximizes the exposure of specific surface area and promotes the adsorption of indicator cations. The equilibrium pH is typically set 1.5 pH units above the point of zero charge (PZC) to ensure a negatively charged surface, facilitating effective cation adsorption. However, both the initial and equilibrium pH should not be excessively high, as elevated pH levels can reduce the sensitivity of ion-selective electrodes and increase measurement complexity [33]. When a large number of unadsorbed indicator cations remain in the system, the accuracy of surface potential measurements is compromised, leading to transfer errors and decreased precision in surface property parameter determinations.

Significant differences in optimal ion concentration ratios for kaolinite and goethite are observed in KOH + Ca(OH)_2_ and KOH + CaCl_2_ mixed systems. Table 5 and Table 7 indicate that for kaolinite, the optimal concentration ratio is *c*_K_:*c*_Ca_ = 2:1 in the KOH + Ca(OH)_2_ system and *c*_K_:*c*_Ca_ = 9:1 in the KOH + CaCl_2_ system. Similarly, Table 9 and Table 11 show that for goethite, the optimal ratio is *c*_K_:*c*_Ca_ = 4:1 in the KOH + Ca(OH)_2_ system and *c*_K_:*c*_Ca_ = 20:1 in the KOH + CaCl_2_ system. These large differences in ion concentration ratios arise because, although K^+^ and Ca^2+^ have similar adsorption capabilities, their interactions vary by system. In the KOH + Ca(OH)_2_ mixture, K^+^ and Ca^2+^ engage in competitive, simultaneous adsorption, making a high concentration ratio unnecessary. However, in the KOH + CaCl_2_ system, K+ first occupies binding sites, and the pH condition required by the system is maintained by OH^−^ from KOH, leading to larger differences in the required concentration ratios for these indicator cations. Both kaolinite and goethite effectively allow surface property parameters to be measured across the K/Ca mixed systems, with slight variations in operational steps and equilibrium time.

For different materials, after determining the appropriate pH conditions, the preferred testing system is selected as a mixed KOH + Ca(OH)_2_ solution with an ion concentration ratio of *c*_K_:*c*_Ca_ = 1:1. The weakly adsorbed ions are identified based on ion adsorption capacity and adsorption ratio. The ion concentration ratio is then gradually expanded (e.g., *c*_K_:*c*_Ca_ = 2:1, *c*_K_:*c*_Ca_ = 4:1, *c*_K_:*c*_Ca_ = 6:1), with subsequent adjustments made according to measurement results to determine surface property parameters. The KOH + CaCl_2_ system offers advantages in terms of easier liquid addition, more efficient control of the ion concentration ratio, and the potential for component-wise addition during continuous measurements. This approach facilitates tracking changes in ion adsorption ratios, ultimately defining the appropriate range of ion concentration ratios.

### 4.2. Sources of Error and Measurement Considerations

The ion-selective electrode method is employed in this experiment to determine surface property parameters using a K/Ca system. During the measurement process, experimental reagents and procedures may introduce errors, which could affect the accuracy of the results. Therefore, it is essential to analyze potential sources of error in the experiment and specify precautions during measurement to enhance the reliability of the data.

(1) In the KOH + Ca(OH)_2_ mixed system, calcium hydroxide exists as a suspension. As a result, uneven distribution of calcium hydroxide may occur when extracting the mixed electrolyte solution. If the solution is not thoroughly homogenized, the actual concentration of Ca^2^⁺ added may differ from the intended concentration, making it difficult for the indicator ions to reach adsorption equilibrium and thus increasing experimental errors. Therefore, when using the KOH + Ca(OH)_2_ mixed electrolyte solution, it is necessary to ensure complete homogenization before sampling. The preparation time of the mixed solution should not exceed 3 h to prevent inadequate uniformity. In contrast, in the KOH + CaCl_2_ system, KOH solution is added first, followed by CaCl_2_ solution, without forming a suspension. This approach improves experimental accuracy.

(2) The sensitivity of the sodium and calcium composite electrodes decreases under excessively high pH conditions, increasing the complexity and potential errors in measurements. Therefore, the pH should be maintained below 10.0. Moreover, an initially high pH may indicate that the amount of indicator ions added exceeds the adsorption capacity, resulting in excessive residual ions in the test solution. According to equation (15), this increases the error in surface potential measurements and imposes stricter requirements on the precision of experimental operations.

(3) When adjusting the pH to the target range using CH_3_COOH, it is recommended to use a gradual, multi-step approach, adding the solution dropwise and uniformly to the surface of the test solution. Uneven or excessive addition can cause non-uniform distribution of H⁺ ions in the solution, leading to rapid pH fluctuations and difficulty in maintaining a stable target range.

(4) During the measurement of the test solution with the ion composite electrode, the immersion position should be kept consistent, along with a uniform stirring speed, to ensure a stable and consistent ion exchange rate within the sensitive membrane of the composite electrode.

## 5. Conclusions

(1) The pH significantly influences the adsorption capacity and adsorption ratio of indicator cations, thereby affecting the accuracy of surface property parameters.

(2) The mode of electrolyte addition and its concentration influence the accuracy of measurements by altering the adsorption state and equilibrium time of the two types of indicator cations.

(3) For kaolinite, the optimal initial pH conditions are 7.5–8.5 in the KOH + Ca(OH)_2_ system and 8.0–8.5 in the KOH + CaCl_2_ system, while the equilibrium pH for both systems is 7.5–8.0. The ideal ion concentration ratios are *c*_K_:*c*_Ca_ = 2:1 and 9:1, respectively.

(4) For goethite, the optimal initial and equilibrium pH conditions are 8.5–9.0 and 7.5–8.0 in both the KOH + Ca(OH)_2_ and KOH + CaCl_2_ systems, with ideal ion concentration ratios of 4:1 and 20:1, respectively.

(5) Further systematic studies are required to better understand the influence of ion concentration ratios on the surface property measurements of variable charge minerals.

## Figures and Tables

**Table 1:** Results of surface property parameters at initial pH = 7.5,8.5, and 9.0 (equilibrium pH = 7.8 ± 0.2).

**Sample**	**Initial pH**	**W** **(g)**	***c*_K_** **(mmol/L)**	***c*_Ca_ (mmol/L)**	**$c_{K}^{0}$ (mmol/L)**	**$c_{\mathrm{Ca}}^{0}$ (mmol/L)**	***n*_K_**	***n*_Ca_**
	7.40	5.0052	2.00	2.00	1.09	0.30	0.547	0.148
H^+^-Kaolinite	7.40	5.0255	2.00	2.00	1.10	0.32	0.548	0.161
	7.50	5.0202	2.00	2.00	1.09	0.32	0.546	0.162
Mean	7.43	5.0170	2.00	2.00	1.09	0.32	0.547	0.157
H^+^-Kaolinite	8.25	5.0189	2.00	2.00	1.25	0.49	0.625	0.245
	8.23	5.0325	2.00	2.00	1.22	0.49	0.612	0.244
	8.21	5.0393	2.00	2.00	1.28	0.52	0.642	0.257
Mean	8.23	5.0302	2.00	2.00	1.25	0.50	0.626	0.249
H^+^-Kaolinite	9.13	5.0309	2.00	2.00	1.45	0.88	0.725	0.439
	9.18	5.0175	2.00	2.00	1.45	0.88	0.724	0.441
	9.23	5.0039	2.00	2.00	1.44	0.90	0.722	0.447
Mean	9.18	5.0174	2.00	2.00	1.45	0.89	0.724	0.443
**Sample**	**N_K_ (cmol/kg)**	**N_Ca_ (cmol/kg)**	***φ*** **_0_** **(V)**	***σ*** **_0_** **(C/m^2^)**	***E*** **_0_** **(10^8^ V/m)**	***T_C_*** **_0_** **(cmol/kg)**	***S*** **(m^2^/g)**	
H^+^-Kaolinite	0.91	1.70	−0.102	0.0544	0.077	4.31	76.5	
	0.90	1.68	−0.098	0.0476	0.067	4.26	86.4	
	0.91	1.68	−0.097	0.0464	0.065	4.26	88.7	
Mean	0.91	1.69	−0.099	0.0494	0.070	4.28	83.9	
Standard Deviation	0.00	0.01	0.002	0.0035	0.005	0.02	5.3	
H^+^-Kaolinite	0.90	1.81	−0.0887	0.0407	0.057	4.53	107.4	
	0.93	1.82	−0.0860	0.0367	0.052	4.56	119.8	
	0.86	1.78	−0.0891	0.0423	0.060	4.43	101.0	
Mean	0.90	1.80	−0.0880	0.0399	0.056	4.51	109.4	
Standard Deviation	0.03	0.01	0.0014	0.0023	0.003	0.06	7.8	
H^+^-Kaolinite	0.77	1.57	−0.0696	0.0254	0.036	3.91	148.3	
	0.77	1.57	−0.0690	0.0249	0.035	3.90	151.3	
	0.78	1.55	−0.0675	0.0237	0.033	3.87	158.0	
Mean	0.77	1.56	−0.0687	0.0247	0.035	3.90	152.5	
Standard Deviation	0.00	0.01	0.0009	0.0007	0.001	0.02	4.1	

Annotation: surface potential (*φ*_0_), surface charge density (*σ*_0_), surface electric field intensity (*E*_0_), surface charge quantity (*T_C_*_0_), and specific surface area (*S*).

**Table 2:** Ion adsorption capacity of goethite at different initial pH.

Initial pH	Equilibrium pH	W (g)	$c_{K}^{0}$(mmol/L)	$c_{\mathrm{Ca}}^{0}$(mmol/L)	N_K_ (cmol/kg)	N_Ca_ (cmol/kg)	*n*_K_	*n*_Ca_
8.0		2.0212	1.66	1.00	1.02	2.99	0.829	0.499
	8.0	2.0100	1.66	0.98	1.02	3.06	0.828	0.486
		2.0154	1.66	1.01	1.00	2.96	0.832	0.503
Mean		2.0155	1.66	1.00	1.01	3.00	0.829	0.496
		2.0147	1.62	0.85	1.42	4.31	0.809	0.421
9.0	9.0	2.0139	1.63	0.93	1.37	3.99	0.815	0.464
		2.0660	1.66	0.94	1.26	3.97	0.830	0.467
Mean		2.0315	1.64	0.91	1.35	4.09	0.818	0.451
		2.0543	1.75	1.09	0.92	3.42	0.876	0.541
9.0	8.0	2.0661	1.75	1.07	0.91	3.46	0.878	0.535
		2.0626	1.77	1.12	0.85	3.29	0.886	0.558
Mean		2.0610	1.76	1.09	0.89	3.39	0.880	0.545

**Table 3:** Surface properties of goethite under different pH conditions.

Initial pH	Equilibrium pH	*φ*_0_ (V)	*σ*_0_ (C/m^2^)	*E*_0_ (10^8^ V/m)	*T_C_*_0_ (cmol/kg)	*S* (m^2^/g)
8.0		−0.0884	0.0550	0.0776	6.99	122.6
	8.0	−0.0904	0.0587	0.0828	7.15	117.5
		−0.0885	0.0553	0.0780	6.92	120.8
Mean		−0.0891	0.0563	0.0795	7.02	120.3
Standard Deviation		0.0009	0.0017	0.0024	0.09	2.1
		−0.0965	0.0698	0.0985	10.04	138.8
9.0	9.0	−0.0903	0.0574	0.0810	9.36	157.4
		−0.0947	0.0680	0.0960	9.21	130.7
Mean		−0.0938	0.0651	0.0918	9.54	142.3
Standard Deviation		0.0026	0.0055	0.0078	0.36	11.2
		−0.0989	0.0856	0.1210	7.76	87.5
9.0	8.0	−0.101	0.0920	0.1300	7.83	82.1
		−0.100	0.0911	0.1290	7.43	78.7
Mean		−0.100	0.0895	0.1260	7.67	82.8
Standard Deviation		0.001	0.0028	0.0040	0.17	3.6

**Table 4:** Ion adsorption capacity of kaolinite at different concentration ratios.

*c*_K_:*c*_Ca_	W (g)	$c_{K}^{0}$ (mmol/L)	$c_{\mathrm{Ca}}^{0}$ (mmol/L)	N_K_ (mol/g)	N_Ca_ (mol/g)	*n*_K_	*n*_Ca_
1:1	5.0052	1.09	0.30	0.91	1.70	0.547	0.148
	5.0255	1.10	0.32	0.90	1.68	0.548	0.161
	5.0202	1.09	0.32	0.91	1.68	0.546	0.162
Mean	5.0170	1.09	0.32	0.91	1.69	0.547	0.157
	5.0277	1.65	0.30	1.35	1.71	0.549	0.150
1.5:1	5.0116	1.75	0.36	1.25	1.65	0.584	0.179
	5.0236	1.72	0.34	1.28	1.67	0.573	0.168
Mean	5.0210	1.71	0.33	1.29	1.68	0.569	0.166
	5.0012	1.76	1.76	1.37	1.54	0.586	0.074
2:1	5.0140	0.11	0.11	1.41	1.54	0.572	0.078
	5.0269	1.72	1.72	1.48	1.54	0.552	0.076
Mean	5.0140	0.12	0.12	1.42	1.54	0.570	0.076

**Table 5:** Surface property parameters of kaolinite at different concentration ratios.

*c*_K_:*c*_Ca_	*φ*_0_ (V)	*σ*_0_ (C/m^2^)	*E*_0_ (10^8^ V/m)	*T_C_*_0_ (cmol/kg)	*S* (m^2^/g)
1:1	−0.102	0.0544	0.077	4.31	76.5
	−0.098	0.0476	0.067	4.26	86.4
	−0.097	0.0464	0.065	4.26	88.7
Mean	−0.099	0.0494	0.070	4.28	83.9
Standard Deviation	0.002	0.0035	0.005	0.02	5.3
	−0.103	0.0562	0.079	4.77	81.9
1.5:1	−0.100	0.0545	0.077	4.55	80.4
	−0.101	0.0562	0.079	4.63	79.5
Mean	−0.101	0.0556	0.078	4.65	80.6
Standard Deviation	0.001	0.0008	0.001	0.09	1.0
	−0.148	0.203	0.287	4.45	21.1
2:1	−0.143	0.169	0.239	4.48	25.6
	−0.140	0.151	0.213	4.56	29.2
Mean	−0.144	0.174	0.246	4.50	25.3
Standard Deviation	0.003	0.0218	0.031	0.04	3.3

**Table 6:** Ion adsorption capacity of kaolinite at different concentration ratios.

*c*_K_:*c*_Ca_	W (g)	$c_{K}^{0}$(mmol/L)	$c_{\mathrm{Ca}}^{0}$ (mmol/L)	N_K_ (mol/g)	N_Ca_ (mol/g)	*n*_K_	*n*_Ca_
7:1	5.0789	3.64	0.13	2.45	0.86	0.659	0.158
	5.0658	3.76	0.13	2.30	0.86	0.681	0.164
	5.0691	3.77	0.12	2.29	0.87	0.681	0.150
Mean	5.0713	3.72	0.12	2.35	0.87	0.674	0.158
	5.0003	3.62	0.08	2.53	0.80	0.649	0.109
8:1	5.0063	3.58	0.07	2.58	0.80	0.641	0.107
	5.0005	3.53	0.07	2.65	0.81	0.632	0.097
Mean	5.0024	3.58	0.07	2.58	0.81	0.641	0.104
	5.0025	3.69	0.06	2.47	0.72	0.656	0.094
9:1	5.0034	3.70	0.06	2.46	0.72	0.658	0.098
	5.0030	3.60	0.06	2.59	0.73	0.639	0.093
Mean	5.0030	3.66	0.06	2.51	0.72	0.651	0.095

**Table 7:** Surface property parameters of kaolinite at different concentration ratios.

*c*_K_:*c*_Ca_	*φ*_0_ (V)	*σ*_0_ (C/m^2^)	*E*_0_ (10^8^ V/m)	*T_C_*_0_ (cmol/kg)	*S* (m^2^/g)
7:1	−0.124	0.0871	0.123	4.18	46.3
	−0.127	0.0985	0.139	4.01	39.3
	−0.132	0.1145	0.162	4.04	34.0
Mean	−0.128	0.1000	0.141	4.08	39.9
Standard Deviation	0.003	0.0112	0.016	0.07	5.0
	−0.143	0.139	0.196	4.13	28.7
8:1	−0.142	0.134	0.190	4.18	30.0
	−0.146	0.148	0.208	4.27	27.9
Mean	−0.144	0.140	0.198	4.19	28.9
Standard Deviation	0.002	0.005	0.008	0.06	0.9
	−0.153	0.178	0.251	3.92	21.3
9:1	−0.151	0.169	0.238	3.90	22.3
	−0.149	0.158	0.222	4.04	24.8
Mean	−0.151	0.168	0.237	3.95	22.8
Standard Deviation	0.001	0.008	0.012	0.06	1.5

**Table 8:** Ion adsorption capacity of goethite at different concentration ratios.

*c*_K_:*c*_Ca_	W (g)	$c_{K}^{0}$ (mmol/L)	$c_{\mathrm{Ca}}^{0}$ (mmol/L)	N_K_ (mol/g)	N_Ca_ (mol/g)	*n*_K_	*n*_Ca_
4:1	2.0374	3.60	0.42	1.47	2.21	0.902	0.413
	2.0136	3.62	0.41	1.41	2.22	0.906	0.410
	2.0025	3.63	0.42	1.35	2.19	0.909	0.417
Mean	2.0178	3.62	0.42	1.41	2.21	0.906	0.413
	2.0031	4.36	0.36	2.04	2.09	0.874	0.360
5:1	2.0063	4.44	0.39	1.76	1.97	0.890	0.391
	2.0514	4.41	0.38	1.90	2.04	0.883	0.374
Mean	2.0203	4.40	0.38	1.90	2.03	0.882	0.375

**Table 9:** Surface property parameters of goethite at different concentration ratios.

*c*_K_:*c*_Ca_	*φ*_0_ (V)	*σ*_0_ (C/m^2^)	*E*_0_ (10^8^ V/m)	*T_C_*_0_ (cmol/kg)	*S* (m^2^/g)
4:1	−0.137	0.238	0.336	5.90	23.9
	−0.140	0.266	0.376	5.86	21.2
	−0.140	0.276	0.390	5.72	20.0
Mean	−0.139	0.260	0.367	5.83	21.7
Standard Deviation	0.002	0.016	0.023	0.08	1.6
	−0.135	0.204	0.288	6.22	29.4
5:1	−0.136	0.223	0.315	5.70	24.7
	−0.136	0.217	0.306	5.97	26.6
Mean	−0.135	0.215	0.303	5.96	26.9
Standard Deviation	0.001	0.008	0.011	0.21	1.9

**Table 10:** Ion adsorption capacity of goethite at different concentration ratios.

*c*_K_:*c*_Ca_	W (g)	$c_{K}^{0}$ (mmol/L)	$c_{\mathrm{Ca}}^{0}$ (mmol/L)	N_K_ (mol/g)	N_Ca_ (mol/g)	*n*_K_	*n*_Ca_
18:1	2.0118	4.88	0.08	3.01	0.78	0.841	0.258
	2.0336	4.87	0.08	3.03	0.78	0.840	0.257
	2.0508	4.91	0.08	2.90	0.79	0.846	0.250
Mean	2.0321	4.89	0.08	2.98	0.78	0.842	0.255
	2.0069	4.93	0.07	2.89	0.73	0.847	0.225
20:1	2.1470	4.90	0.06	2.98	0.74	0.842	0.221
	2.1123	4.87	0.06	3.1	0.74	0.836	0.221
Mean	2.0887	4.90	0.06	2.99	0.73	0.841	0.222

**Table 11:** Surface property parameters of goethite at different concentration ratios.

*c*_K_:*c*_Ca_	*φ*_0_ (V)	*σ*_0_ (C/m^2^)	*E*_0_ (10^8^ V/m)	*T_C_*_0_ (cmol/kg)	*S* (m^2^/g)
18:1	−0.145	0.155	0.219	4.56	28.4
	−0.144	0.154	0.218	4.59	28.7
	−0.149	0.177	0.250	4.48	24.4
Mean	−0.146	0.162	0.229	4.54	27.2
Standard Deviation	0.002	0.0106	0.015	0.05	2.0
	−0.156	0.210	0.297	4.36	20.0
20:1	−0.155	0.204	0.287	4.45	21.1
	−0.153	0.187	0.264	4.57	23.6
Mean	−0.154	0.200	0.283	4.46	21.5
Standard Deviation	0.001	0.0097	0.014	0.09	1.5

## Data Availability

The original contributions presented in the study are included in the article, further inquiries can be directed to the First author.

## Appendix A

### Appendix A.1. BET Analysis Results of Kaolinite

The kaolinite used in this experiment is characterized using the surface area and pore size distribution analyzer at the School of Chemistry and Chemical Engineering, Southwest University. The specific surface area is determined to be 17.501 m^2^/g.

### Appendix A.2. BET Analysis Results of Goethite

The goethite used in this experiment is analyzed using a surface area and pore size distribution analyzer at the School of Chemistry and Chemical Engineering, Southwest University. The specific surface area is measured to be 19.492 m^2^/g.
